# Global Burden of Human Mycetoma: A Systematic Review and Meta-analysis

**DOI:** 10.1371/journal.pntd.0002550

**Published:** 2013-11-07

**Authors:** Wendy W. J. van de Sande

**Affiliations:** ErasmusMC, Department of Medical Microbiology & Infectious Diseases, Rotterdam, The Netherlands; University of California San Diego School of Medicine, United States of America

## Abstract

Mycetoma is a chronic infectious disease of the subcutaneous tissue with a high morbidity. This disease has been reported from countries between 30°N and 15°S since 1840 but the exact burden of disease is not known. It is currently unknown what the incidence, prevalence and the number of reported cases per year per country is. In order to estimate what the global burden of mycetoma is, a meta-analysis was performed. In total 50 studies were included, which resulted in a total of 8763 mycetoma cases. Most cases were found in men between 11 and 40 years of age. The foot was most commonly affected. Most cases were reported from Mexico, Sudan and India. *Madurella mycetomatis* was the most prevalent causative agent world-wide, followed by *Actinomadura madurae*, *Streptomyces somaliensis*, *Actinomadura pelletieri*, *Nocardia brasiliensis* and *Nocardia asteroides*. Although this study represents a first indication of the global burden on mycetoma, the actual burden is probably much higher. In this study only cases reported to literature could be used and most of these cases were found by searching archives from a single hospital in a single city of that country. By erecting (inter)national surveillance programs a more accurate estimation of the global burden on mycetoma can be obtained.

## Author Summary

Mycetoma is a chronic infection resulting in large masses of the subcutaneous tissue of mainly the foot. It can be caused by bacteria or fungi. Treatment for most mycetoma cases is poor and amputations are common. Although this disease was already described almost 200 years ago, it is currently not known how many people over the globe actually suffer from this disease and which countries are mostly affected. These data are useful because they can be used to concentrate medical help in places where it is really needed and to focus to search on new medication on the most common causative agents. Since mycetoma is not a reportable disease, a meta-analysis was performed based from reports in literature, in order to estimate what the global burden of mycetoma is. In total 8763 mycetoma cases were analysed. It appeared that most cases were reported from Mexico, Sudan and India and that the fungus *Madurella mycetomatis* was the most prevalent causative agent world-wide. Although this study represents a first indication of the global burden on mycetoma, the actual burden is probably much higher. By erecting (inter)national surveillance programs a more accurate estimation of the global burden on mycetoma can be obtained.

## Introduction

Mycetoma is a chronic infectious disease, characterized by the formation of tumor like-swellings and the formation of grains. This disease usually begins with a small trauma on the foot which introduces the causative agent into the subcutaneous tissue. Inside this tissue the causative agent will organize itself into small granules called grains. A small nodule will arise which gradually will grow into a large subcutaneous mass with sinuses which will secrete pus and grains. Eventually the bone will be invaded and a large mutilating lesion will be formed and the foot will be amputated [1]. Although mycetoma is usually found in the foot, other body sides can be affected as well [2].

Although, the first documented clinical cases of mycetoma were already described in 1842 by Gill, uncertainty remains about the total number of cases world-wide as already mentioned by Gokhale in 1981 [3]. A first attempt to map the number of mycetoma cases in a certain region was made by Abbott in the early 1950s [4]. Abbott, studied 1321 mycetoma cases in the Sudan in a 2.5 year period and published his findings in 1956 [4]. Many scientists were surprised by the amount of cases to be retrieved in such a small period, which could indicate that the burden of mycetoma was higher than previously thought. Therefore, others started to determine the burden of mycetoma in other countries known to be endemic for mycetoma, such as Congo [5], Somalia [6], [7], [8], [9], Argentina [10], [11] and Mexico [12], [13]. Although several studies have been performed to determine the prevalence of mycetoma in a certain region or country, no overall study has been performed to determine the prevalence of mycetoma world-wide. Furthermore mycetoma is not a reportable disease; therefore it is currently still not known what the global burden of mycetoma is.

In the surveys performed in the past and based on case-studies it appeared many different species, both bacteria and fungi, are able to cause mycetoma. In the review written by Ahmed et al. 48 species were listed as causative agents [1]. Some of these agents were considered to be common causative agents of mycetoma while others were found only rarely. A definition on common or rare was not given. Recently, based on sequencing either the 16S region (for bacteria) or the ITS region (for fungi) more causative agents were added to this list [14], [15], [16]. Since molecular identification is not used in the endemic regions, the total number of species able to cause mycetoma is still not known. Furthermore, no clear definition is given which species are common causative agents of human mycetoma and which are only rarely implicated.

The distribution of the mycetoma causative agents is not equal around the globe. In overall, actinomycetoma – mycetoma caused by bacteria – is more commonly found in Middle and South-America while eumycetoma – mycetoma caused by fungi – is more commonly found in Africa [4], [13]. But within a country, this could also differ per region [17], [18].

In order to estimate what the true burden of mycetoma is globally, a meta-analysis was performed in which all studies in which the epidemiology of the mycetoma causative agents was studied were reviewed. Studies with more than 10 cases were included. The burden of mycetoma was determined in terms of prevalence and the number of reported cases per year per country. Furthermore we determined which species were most commonly associated with mycetoma and prepared definitions based on the ∼8000 cases to determine which species were commonly, occasionally or rarely associated with mycetoma development.

## Methods

### Search strategy

A systematic review of available literature on the epidemiology of mycetoma was searched using the electronic database PubMed with the use of the following search terms: Mycetoma AND epidemiology or Madura foot AND epidemiology. Studies published in languages other than English, French, Spanish, Portuguese, German or Dutch were excluded. The search was supplemented by reviewing the reference lists of all selected studies. Studies were excluded if the number of patients studied was <10 patients, if a study was already published before and if the paper was written as a review.

### Prevalence

To determine the prevalence for each country, the number of reported cases for each year for that country were divided through the total population of that country for that year. Population figures were derived from IndexMundi (http://www.indexmundi.com/facts/indicators/SP.POP.TOTL/compare#country=ma).

This site only gives data from 1960 onwards. If studies had data from older years, the population size of 1960 was used to calculate the prevalence. To determine the prevalence within individual Indian states, population figures were derived from http://www.citypopulation.de/India.html.

### Calculations and definitions to determine prevalent species

From each study, the sampling period, the region of sampling, the sex distribution, age distribution and species isolated were recorded. To determine which species were most prevalent the percentages of each species present in a certain study was calculated. The percentage was used for comparison since some studies reported on >2000 cases while others reported only 11. To determine the global prevalence, the sum of the means for each country for a certain species was taken and then divided by the total of countries included. To determine which species were most prevalent the following definitions were used: common: >5% of the reported cases world-wide was caused by this species; occasional: 1–5% of the reported cases world-wide was caused by this species; rare: <1% of the reported cases world-wide was caused by this species.

## Results

**Figure 1:**
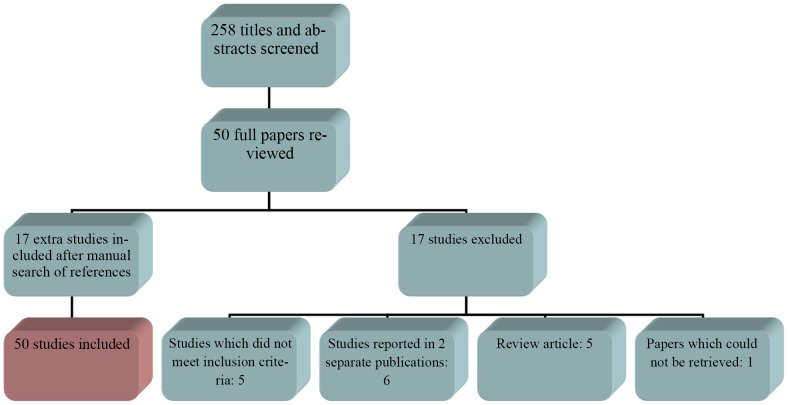
Flow diagram of literature search. In this flow diagram it is shown that 258 titles and abstracts were screened initially. 50 papers were screened of which 17 were excluded due to failing the inclusion criteria, studies reported in two different studies, being reviews and 1 could not be retrieved. After manually searching the references of each of the retrieved papers, 17 more papers were identified and included in the analysis. This resulted in a total of 50 papers analysed.

Our systematic review identified 258 studies of which 49 full articles were reviewed (Figure 1). Via manually searching of the references used in those studies an additional 17 studies were included. In total 17 studies were excluded for analysis because they did not meet the inclusion criteria (5 studies) [11], [19], [20], [21], [22], or contained data already published in another paper (6 studies) [8], [23], [24], [25], [26], [27] or consisted of reviews instead of original data (5 studies) [3], [5], [28], [29], [30] or could not be retrieved (1 study). This led to a total number of 50 studies to be included into the study [4], [6], [7], [9], [10], [12], [13], [17], [18], [31], [32], [33], [34], [35], [36], [37], [38], [39], [40], [41], [42], [43], [44], [45], [46], [47], [48], [49], [50], [51], [52], [53], [54], [55], [56], [57], [58], [59], [60], [61], [62], [63], [64], [65], [66], [67], [68], [69], [70], [71]. From each of these studies the number of cases was recorded, the gender of the patients, age of the patients, site of the lesion and the causative agents isolated.

### World-wide distribution of mycetoma

In total 8763 mycetoma cases were included in this study. Most of the cases were reported from Mexico (2607 cases) [12], [13], [53], Sudan (2555 cases) [4], [34], [70] and India (1392 cases) [17], [18], [35], [42], [44], [51], [59], [60], [65], [66], [67]. Countries with only limited cases reported are Uganda (11 cases) [64], Rumania (13 cases) [68], Nigeria (15 cases) [55], Bulgaria (16 cases) [58] and Thailand (17 cases) [56].

**Figure 2:**
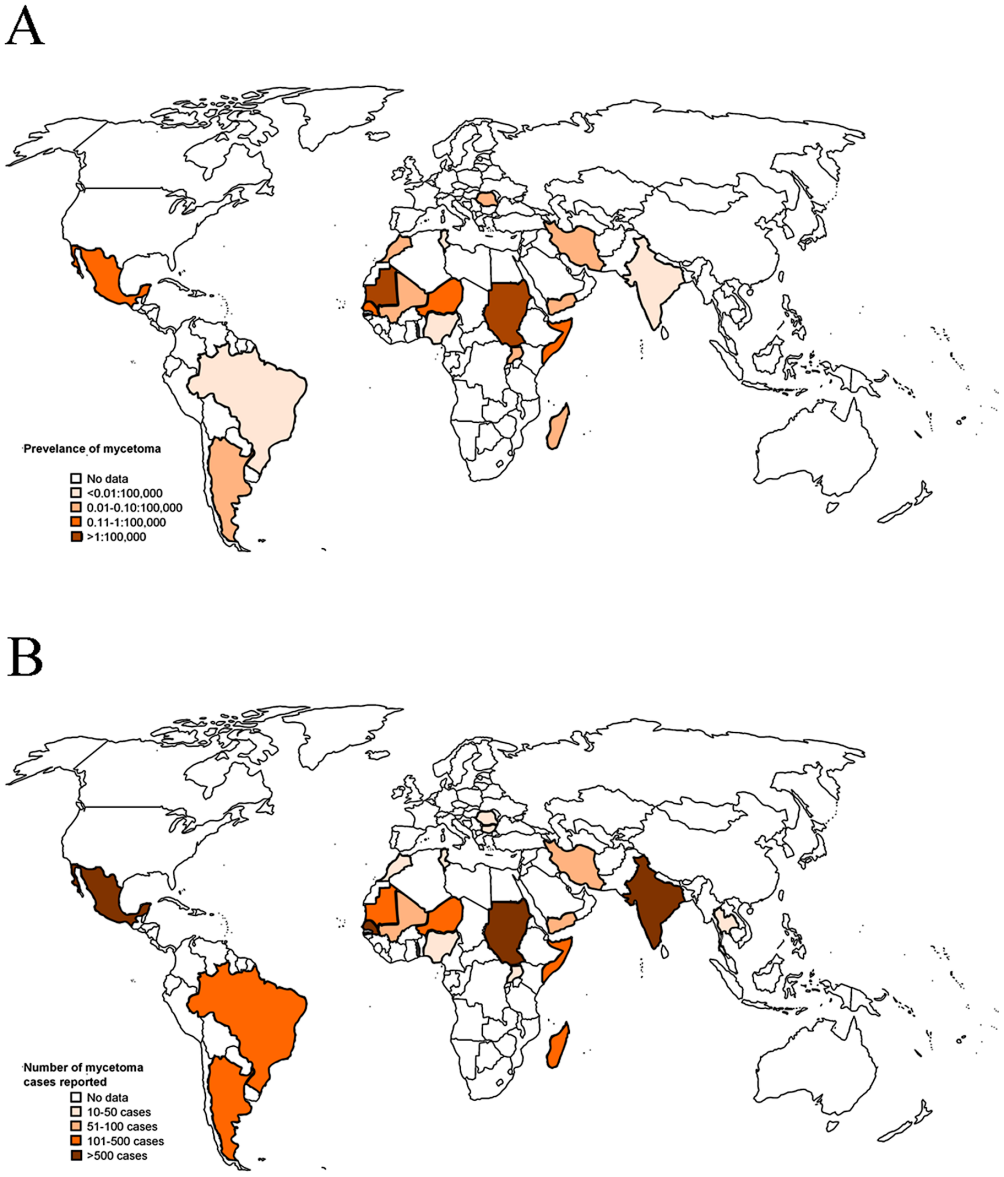
Prevalence and the number of reported cases of mycetoma. A: Average prevalence of mycetoma cases as calculated by the number of cases reported in a year in a certain country divided trough the total population of that country of that same year as reported by www.indexmundi.com/facts/indicators/SP.POP.TOTL/compare. B: the average number of mycetoma cases reported per year per country.

In order to estimate the prevalence of mycetoma the number of reported cases for each year for that country were divided through the total population of that country for that year as shown in figure 2A. As you can see in this figure, countries with the highest prevalence include Mauritania (prevalence of 3.49 cases per 100,000 inhabitants) and Sudan (prevalence of 1.81 cases per 100,000 inhabitants). Also Mexico, Senegal, Niger and Somalia have a relatively high prevalence for mycetoma. In order estimate the number of mycetoma cases reported per year the following calculation was made: the total number of cases was divided through the number of years in which they were gathered (figure 2B). So if we take as an example Mali. In Mali 54 cases were reported in a 10 year period between 1985 and 1994 [45]. So in total there were 54/10 = 5.4 cases/year seen. According to figure 2B, Sudan reported the highest number of cases yearly, namely 106 per year. For Mexico and Mauritania, this was 80.7 and 69.7 respectively.

### Mycetoma is commonly found in the foot of man

**Figure 3:**
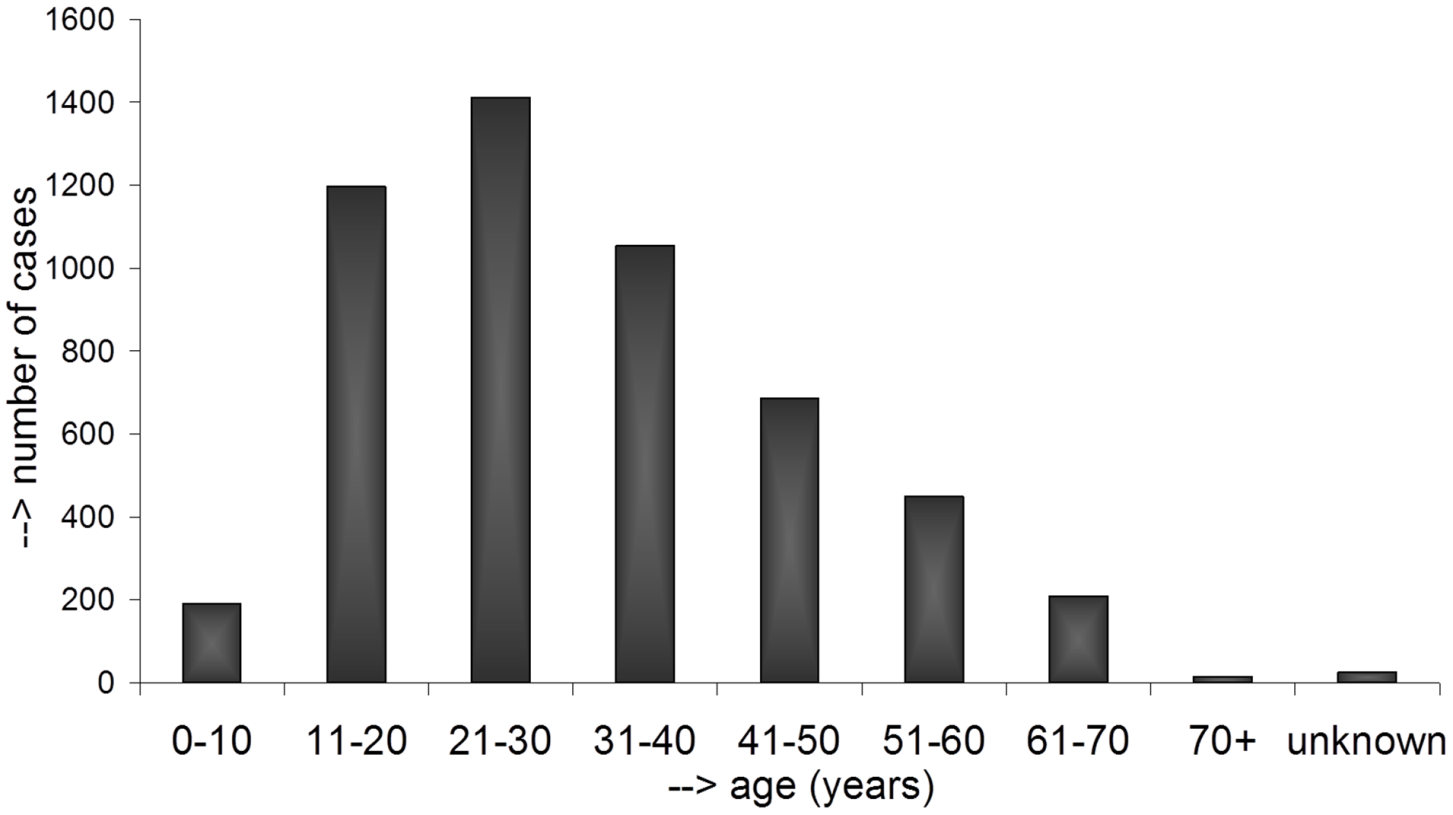
Age distribution of the mycetoma patients. Age distribution as reported in 5240 cases of mycetoma. For the other 3523 cases no detailed information was available.

**Figure 4:**
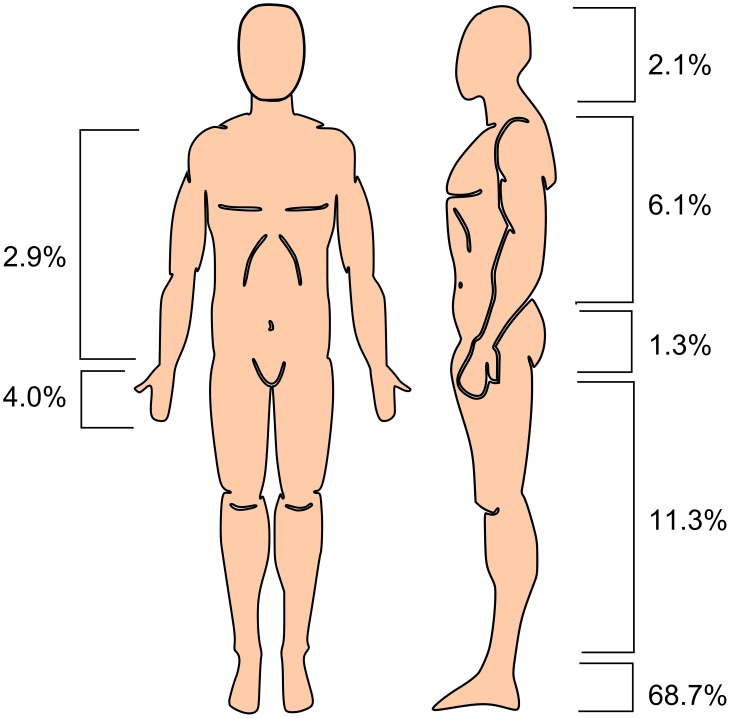
Mycetoma lesion site. The mycetoma lesion site. In this figure the percentage of cases reported from a certain body site is shown. For 2.6% of the cases the lesion site was unknown to the authors and 1.0% of the patients had multiple lesions. The percentages shown in this figure were calculated from data obtained from 4581 cases. For the other 4182 cases no detailed information was available.

World-wide most cases were found in men: 4060 cases in men versus 1175 cases in women. Exceptions were Thailand and Tunisia. In Thailand, men and women were equally affected (8 man and 9 women), while in Tunisia actually mycetoma was reported more in women (16 cases) than in men (12 cases). As is seen in figure 3, of the 5240 cases in which age was reported, 70% of the cases (3664 cases) were found in people with an age between 11 and 40 (3664 cases). Furthermore the most affected body site was the foot (68.7%), followed by the leg (9.9%), trunk (6.1%) and arm (4.0%) (Figure 4). Although these observations were based on all the cases reported world-wide, there are some regional differences. In all studies, the foot was the most reported lesion site, but in South-American patients the trunk was more often the site of infection than in African or Asian patients. For example, in Sudan the trunk as lesion site was only reported in 1.4% of the cases, while in Mexico it was reported in 18.7% of all cases.

### The most common causative agents

As is seen in table 1, most cases were actinomycetoma cases, but the total number of actinomycetes able to cause mycetoma is less. In total, 7 different actinomycetes were identified in the studied actinomycetoma cases, but for some cases the causative agents was not identified to the species level. Some were classified as *Nocardia* spp. and others as actinomycetoma spp. Based on our criteria the species *Actinomadura madurae*, *Streptomyces somaliensis*, *Actinomadura pelletieri*, *Nocardia brasiliensis* and *Nocardia asteroides* were considered to be common causative agents. Each of them was found in >5% of the studied cases. *Nocardia otidiscaviarum* was only found occasionally, and *Actinomyces israeli* rarely.

**Table 1:** Number of species identified by selected papers.

Species	Colour of the grain*	Number of isolates	Percentage	Prevalence**
**Actinomycetoma**		**4832**	**50.84**	
*Actinomadura madurae*	W/Y/P	594	10.21	C
*Streptomyces somaliensis*	Y/Br	677	10.16	C
*Actinomadura pelletieri*	R	594	6.71	C
*Nocardia brasiliensis*	W	1946	5.78	C
*Nocardia asteroides*	W	108	5.58	C
*Nocardia otidiscaviarum* (synonym: *Nocardia caviae*)	W/Y	51	1.08	O
*Actinomyces israeli*	W/Y	1	0.07	R
*Nocardia* spp	W/Y	371	4.64	
Unidentified actinomycetes		590	6.60	
**Eumycetoma**		**2704**	**41.72**	
*Madurella mycetomatis*	B	2032	26.44	C
*Scedosporium boydii* (synonym: *Scedosporium apiospermum* or *Pseudallescheria boydii*)	W	83	3.52	O
*Falciformispora senegalensis* (synonym: *Leptosphaeria senegalensis*)	B	167	2.01	O
*Trematosphaeria grisea* (synonym: *Madurella grisea*)	B	116	1.62	O
*Acremonium falciforme* (synonym: *Cephalosporium falciforme*)	W	3	0.73	R
*Aspergillus fumigatus*		2	0.40	R
*Exophiala jeanselmei*	B	9	0.37	R
*Geotrichum candidum*		1	0.33	R
*Neotestudina rosatii*	W	4	0.33	R
*Medicopsis romeroi*	B	22	0.26	R
*Medicopsis romeroi* or *Biatriospora mackinnonii* (synonym: *Pyrenochaeta* spp)	B	7	0.21	R
*Aspergillus flavus*	G	2	0.07	R
*Microsporum audouini*	W	1	0.04	R
*Cochliobolus lunatus* (synonym: *Curvularia lunata*)	B	2	0.01	R
*Rhinocladiella atrovirens*		2	0.01	R
*Aspergillus nidulans*	W	1	0.003	R
*Neoscytalidium dimidiatum*		1	0.003	R
*Fusarium* spp	W	13	0.34	
*Cladosporium* spp		1	0.14	
*Exophiala* spp	B	2	0.006	
Unidentified fungi		220	4.72	
**Unknown etiology**		217	7.49	
**TOTAL**		**7753**	**100**	

**<?ENTCHAR ast?>:** colour of the grain: black (B), brown (Br), green (G), pink (P), red (R), white (W), yellow (Y).

**<?ENTCHAR ast?><?ENTCHAR ast?>:** prevalence: common (C): species isolated >5%; occasional (O): species isolated 1–5%; rare species isolated <1%.

For the eumycetoma cases, 18 different fungal species were identified as causative agents. Furthermore, for some cases the fungus could not be identified to the species level, but only to the genus level. These belonged to the genera *Fusarium*, *Cladosporium* or *Exophiala*. For 219 fungi, the fungus was not even identified to the genus level. Only the fungus *Madurella mycetomatis* was considered to be a common causative agent. In fact it appeared the most common causative agent of all mycetoma cases since 24.3% of all studied cases were caused by this fungus. *Scedosporium boydii*, *Falciformispora senegalensis* and *Trematosphaeria grisea* were occasionally isolated from mycetoma cases, and *Acremonium falciforme*, *Aspergillus fumigatus*, *Exophiala jeanselmei*, *Geotrichum candidum*, *Neotestudina rosatii*, *Medicopsis romeroi*, *Biatriospora mackinnonii*, *Aspergillus flavus*, *Microsporum audouini*, *Cochliobolus lunatus*, *Rhinocladiella atrovirens*, *Aspergillus nidulans* and *Neoscytalidium dimidiatum* only rarely.

**Figure 5:**
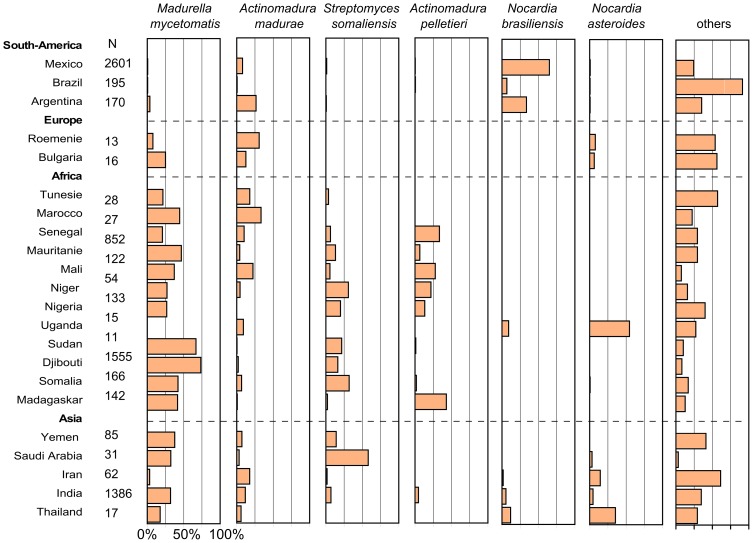
Distribution of the most common causative agents per country. The distribution of *Madurella mycetomatis*, *Actinomadura madurae*, *Streptomyces somaliensis*, *Actinomadura pelletieril*, *Nocardia brasiliensis* and *Nocardia asteroids* per country. For each country the number (N) of species identified is given. The percentage of *Madurella mycetomatis*, *Actinomadura madurae*, *Streptomyces somaliensis*, *Actinomadura pelletieril*, *Nocardia brasiliensis* and *Nocardia asteroids* were calculated from these data and displayed in the corresponding panels.

The identified species were not evenly distributed throughout the world (Figure 5). *M. mycetomatis*, *S. somaliensis* and *A. pelletieri* were highly prevalent in Africa and Asia but hardly found in South-America. In contrast, the most prevalent species encountered in South America, *N. brasiliensis*, was hardly found in Africa, Europe and Asia. The only species which was found on all continents in equal amount was *A. madurae*.

### India, as example

**Figure 6:**
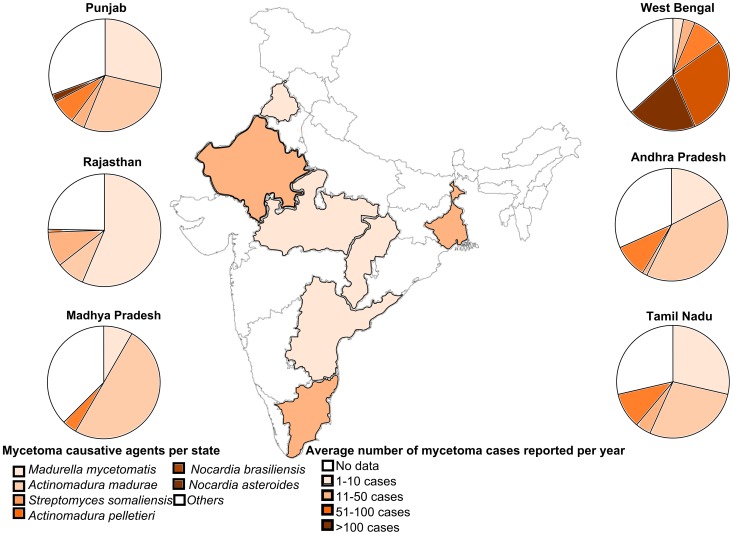
India. Average number of mycetoma cases reported per year in India per state and the causative agents per state.

Until now, we generalized all studies per country, but within a country there can be large differences. As an example India is taken, since from this country we had data from 11 different studies. These studies originated from different parts of the country, namely Punjab, Rajasthan, Madhya Pradesh, Adhra Pradesh, Tamil Nadu and West-Bengal. Most of these states have roughly the same population size at the time most studies were performed (∼50,000,000 inhabitants/state in 1981), only Punjab had a smaller population (16,788,915 inhabitants/state in 1981). As is seen in figure 6, in Rajasthan most cases were reported per year (33.3), followed by Tamil Nadu (16.8 reported cases/year) and West-Bengal (13.2 reported cases/year). The reported species per state also differed, in Rajasthan 62.5% of all mycetoma cases was caused by fungi, while in the other states most cases were caused by actinomycetoma's (54.3%–83.3% of all mycetoma cases). Not surprisingly, the species encountered also differed per state (figure 6).

## Discussion

Since it is currently not known what the global burden of mycetoma is in terms of prevalence and incidence, we tried to make a rough estimation by performing a meta-analysis on the published literature from 1955 onwards. The number of papers published around the globe was relatively little. Only 50 papers could be used, which resulted in a total number of 8763 mycetoma cases documented since 1944. The globally reported number of cases is therefore 127 cases/year. The 8763 mycetoma cases were reported from 23 different countries. For some countries we only had data on 11 cases (e.g. Uganda), while for others we had data on 2607 cases (Mexico). The more cases reported per country the more reliable the data are. Since for most studies we knew in which years the cases were seen, a rough prevalence figure was calculated by dividing the number of cases seen in each year through the total population of that country in that year. This resulted in prevalence numbers ranging from <0.01 cases per 100,000 inhabitants (several countries) to 1.8 cases per 100,000 inhabitants (Sudan) and an average of cases reported per year ranging from 0.9 (Tunesia) to 106 (Sudan) (Figures 2A and 2B). These numbers are gross underestimations of the true prevalence, since only cases reported to literature could be used. Most of these cases were found by searching archives from a single hospital in a single city of that country. Only those studies which searched multiple centres throughout the country, such as the studies performed by Abbott in Sudan [4] and by Lopez Martinez in Mexico [13], more than 1000 patients were documented. Based on these short-comings we estimate that the total number of mycetoma cases will be much higher.

Some evidence of a higher prevalence exists, although not reported to literature. For instance, in 1991 the Mycetoma Research Centre in Khartoum, Sudan was established. Since its founding, 6334 patients have been seen. In 2012 only, 5158 patients were seen of which 402 were new cases. This means that there were (5158 cases/year) seen in Sudan only, which were not reported to literature (prof A. Fahal, personal communication). Furthermore, the incidence calculated on the figures from the Mycetoma Research Centre are most probably also an underestimation, since a cohort study performed in the endemic village Abu Gumri in Sudan performed in 1960, indicated that there was a prevalence of 6.2 per 1000 inhabitants in that particular village [72].

Although the reported number of cases seen per year in this study is an underestimation, they are comparable with the number of cases reported for Buruli Ulcer and human African trypanosomiasis in many countries [73]. For instance, the number of reported Buruli Ulcer cases in 2009 were below 100 cases for Australia, Sudan, Gabon, Nigeria, and Guinea, while there were between 100 and 500 cases reported in Cameroon, Congo and the Democratic republic for the Congo in that year [73]. The number of reported Human African Trypanosomiasis (HAT) from surveillance studies in 2009 were below 100 reported cases for Kenya, Tanzania, Malawi, Zambia, Zimbabwe, Congo, Cameroon and Gabon [74]. The countries in which between 100 and 500 HAT cases were reported included Sudan, Angola and Uganda [74]. The data obtained for both Buruli Ulcer and HAT were from surveillance data, which makes these numbers more reliable than, the number of mycetoma cases/year reported here were derived from single center studies, and represent an underestimation. Therefore the prevalence of mycetoma is probably higher than that of either Buruli Ulcer or HAT. Still, Buruli Ulcer and HAT are reported on the list of neglected diseases while mycetoma is not.

In this study it was shown that mycetoma could be caused by many different micro-organisms, both bacteria and fungi. Globally, most cases were caused by bacteria (50.8%) and a smaller percentage by fungi (41.7%) (Table 1), although this differed per country. In many African countries actually more eumycetoma cases were found as actinomycetoma cases. In table 1 it was also shown that there are many different bacteria and fungi able to cause mycetoma. There were 7 different bacterial species and 16 different fungal species reported. The total list of species able to cause mycetoma is actually longer. Since we only focused on studies reporting more than 10 cases per study, we missed the many species named in case reports. Other bacterial species implicated in mycetoma are *Actinomadura latina*, *Gordonia terrae*, *Nocardia farcinica*, *Nocardia harenae*, *Nocardia mexicana*, *Nocardia transvalensis*, *Nocardia veterana*, *Nocardia yamanashiensis*, *Nocardiopsis dassonvillei* and *Streptomyces sudanensis* [1], [14], [75], [76], [77], [78], [79], [80], [81], [82], [83]. Other fungal species implicated in mycetoma are *Acremonium recifei*, *Cladophialophora bantiana*, *Corynespora cassiicola*, *Curvularia geniculata*, *Diaporthe phaseolorum*, *Fusarium oxysporum*, *Gibberella fujikuroi* (synonym: *Fusarium monoliforme*), *Haematonectria haematococca* (synonym: *Fusarium solani*), *Ilyonectria destructans* (synonym: *Cylindrocarpon destructans*), *Falciformispora tompkinsii* (synonym: *Leptosphaeria tompkinsii*), *Madurella fahalii*, *Madurella pseudomycetomatis*, *Madurella tropicana*, *Microsporum canis*, *Phaeoacremonium parasiticum*, *Phialophora cyanescens* (synonym: *Cylindrocarpon cyanescens*), *Phialophora verrucosa*, *Pleurostomophora ochracea*, *Pseudochaetosphaeronema larense*, *Rhinocladiella atrovirens*, *Sarocladium kiliense* (synonym: *Acremonium kiliense*), *Setosphaeria rostrata* (synonym: *Exserohilum rostrata*) [1], [15], [16], [84], [85], [86], [87], [88], [89], [90], [91], [92], [93], [94], [95], [96], [97], [98]. Since most of these species are only found in case studies, these are probably only rarely associated with mycetoma, if associated at all.

Although most species represented in Table 1 were isolated from multiple cases, some species were only isolated from 1–5 cases, which makes one wonder if this was the true causative agent. Only for *Nocardia asteroides* [99], *Nocardia brasiliensis* [99], [100], [101], [102], *Nocardia caviae* [99], [100], *Nocardia transvalensis* [103], *Madurella mycetomatis* [104], [105] the pathogenicity in animal models has been demonstrated. In all these animal models pathology resembling mycetoma and the formation of grains was demonstrated. The only attempt ever recorded for *Actinomadura madurae*, *Actinomadura pelletieri* and *Streptomyces somalienis* failed [104]. So, one could wonder if all species listed in Table 1 and in the discussion are true causative agents for mycetoma.

The causative agents presented in Table 1 were identified based on histology only or on histology combined with culture. Therefore, the possibility arises that some of the causative agents were misidentified. Although widely used, the limitations of histological diagnosis have been known since the early 1960s. It is impossible to differentiate *Nocardia* spp. to the species level based on histology only, all species form white to yellow, small spherical grains [106], [107]. Also *S. boydii*, *Acremonium* spp and *Fusarium* spp are difficult to differentiate [106], [107]. These species produce white to yellow grains with a dense mass of slender, septate, hyaline hyphae with occasional vesicles or swollen hyphae [107]. The black-grain causative agents *F. senegalensis*, *F. tompkinsii*, *M. romeroi*, *Exophiala jeanselmei* and *T. grisea* also produce similar type of grains [107]. These species produce black grains, which can be tubular or hollow with a darker periphery [107]. The *Falciformispora* grains usually contain larger vesicles than the other species [107]. Some causative agents can cause multiple grain types in histological slides. *Madurella mycetomatis* is known to produce three structural forms of the fungal grain: the filamentous type, the vesicular type and a mixture of both [2]. In the vesicular type of grain the center is light colored and the hyphae in the periphery are brown, just as the ones described above for *F. senegalensis*, *F. tompkinsii*, *M. romeroi*, *Exophiala jeanselmei* and *T. grisea* [2]. For some of the studies culturing of the causative agents was also included. But identification of species based on culturing only, has known to be troublesome too, especially for the fungi. Identification of the fungi is usually achieved by observation of the growth rate, colony morphology, production of conidia and assimilation patterns. Most of the black-grain eumycetoma causative agents only rarely produce conidia and therefore misidentifications are known to occur frequently [108]. A well-known example is *T. grisea*, previously known as *Madurella grisea*. Due to the numerous misidentifications McGinnis suggested in 1996 that this species should be considered a complex of different fungi, classified together because of their sclerotial color and architecture and colony characteristics [109]. Some isolates of *B. mackinnonii* are known to be misidentified as *T. grisea*, since some of these isolates did not form pycnidia at standard media, but only after stimulation [109]. Furthermore, with classical culture methods, not all species can be differentiated. An example is *Streptomyces sudanensis*, which morphologically is equal to *Streptomyces somaliensis*. Only by sequencing the 16S gene of these species, it appeared that out of 9 previously identified *S. somaliensis* strains, 5 were actually misidentified and were renamed *S. sudanensis* [14]. The same was true for *M. mycetomatis*, in 2012 it appeared that *M. mycetomatis* had some close relatives which could only be identified by ITS sequencing, not on morphology [15]. Even when the species was already known, misidentifications have occurred in the past. *N. brasiliensis* has always been considered the most common causative agent of actinomycetoma in Mexico. Sánchez-Herrara demonstrated in 2012, that from the 18 previously identified *N. brasiliensis* isolates obtained from mycetoma patients in Mexico City between 1947 and 1959, only 7 were *N. brasiliensis*, the other 11 were proven to be *N. farcinica* based on sequencing and phenotypic profiles [110]. Therefore it remains doubtful that the data derived from the studies used for this meta-analysis were based on correctly identified species.

Even with all these short-comings listed of this meta-analysis, it still gives a good overview of our current knowledge on the burden of mycetoma world-wide. In order to better estimate the burden, global surveillance programs should be erected, like for instance the surveillance program for Buruli Ulcer. This program is the result of the Global Buruli Ulcer initiative established in 1998 by the WHO and recognized by the 57th World Health Assembly in 2004 [111]. For this surveillance program, standardized forms (BU02) were generated which are now used in the endemic countries. If such a system was developed for mycetoma, better understanding on the global burden, and the epidemiology of this disease will be gained.

## Supporting Information

Checklist S1 PRISMA checklist.
(DOC)

Diagram S2 PRISMA flow chart.
(DOC)
