# Supplementary material for: Global Burden of Human Mycetoma: A Systematic Review and Meta-analysis
Source: PLoS Negl Trop Dis. 2013 Nov 7;7(11):e2550. doi: 10.1371/journal.pntd.0002550 (PMC3820768; doi:10.1371/journal.pntd.0002550)
Supplement: Diagram S2 — PRISMA flow chart. (DOC) [file pntd.0002550.s002.doc]

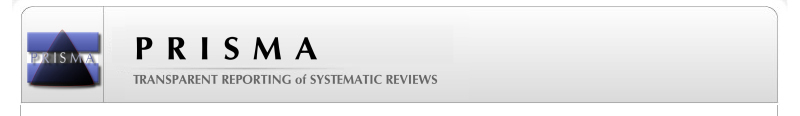
**PRISMA 2009 Flow Diagram**

**Screening**

**Included**

**Eligibility**

**Identification**

Records identified through database searching
(n =258)

Additional records identified through other sources
(n =17)

Records after duplicates removed
(n =275)

Records screened
(n =275)

Records excluded
(n = 0 )

Full-text articles assessed for eligibility
(n = 67 )

Full-text articles excluded, with reasons
(n = 17)

Studies included in qualitative synthesis
(n =50)

Studies included in quantitative synthesis (meta-analysis)
(n = 50)
